# (1 + *u*)-Constacyclic codes over *Z*_4_ + *uZ*_4_

**DOI:** 10.1186/s40064-016-2717-0

**Published:** 2016-08-11

**Authors:** Haifeng Yu, Yu Wang, Minjia Shi

**Affiliations:** 1 Department of Mathematics and Physics, Hefei University, Hefei, China; 2 School of Mathematical Sciences, Anhui University, Hefei, China

**Keywords:** Cyclic code, Constacyclic code, Quasi-cyclic code, Gray map

## Abstract

Constacyclic codes are an important class of linear codes in coding theory. Many optimal linear codes are directly derived from constacyclic codes. In this paper, (1 + *u*)-constacyclic codes over *Z*_4_ + *uZ*_4_ of any length are studied. A new Gray map between *Z*_4_ + *uZ*_4_ and *Z*_4_^4^ is defined. By means of this map, it is shown that the *Z*_4_ Gray image of a (1 + *u*)-constacyclic code of length n over *Z*_4_ + *uZ*_4_ is a cyclic code over *Z*_4_ of length 4*n*. Furthermore, by combining the classical Gray map between *Z*_4_ and *F*_2_^2^, it is shown that the binary image of a (1 + *u*)-constacyclic code of length *n* over *Z*_4_ + *uZ*_4_ is a distance invariant binary quasi-cyclic code of index 4 and length 8*n*. Examples of good binary codes are constructed to illustrate the application of this class of codes.

## Background

Recently, several new classes of rings have been studied in connection with coding theory. Many optimal binary linear codes have been obtained from codes over these rings via some Gray map. In Yildiz and Karadenniz (2010a, b), the authors introduced the ring *F*_2_ + *uF*_2_ + *vF*_2_ + *uvF*_2_ and discussed linear and self-dual codes over *F*_2_ + *uF*_2_ + *vF*_2_ + *uvF*_2_. Later, the structures of cyclic codes and (1 + *u*)-constacyclic codes over *F*_2_ + *uF*_2_ + *vF*_2_ + *uvF*_2_ were studied and many optimal binary linear codes were constructed from such codes in Yildiz and Karadenniz (2011a, b). More generally, cyclic codes over the ring *R*_*k*_ were investigated in Dougherty et al. (2012). Although the rings mentioned above are not finite chain rings, they have rich algebraic structures and produce binary codes with large automorphism groups and new binary self-dual codes. This demonstrates that linear codes over such non-chain rings have been received increasing attention (see Dougherty et al. 2012; Kai et al. 2012; Shi 2014; Shi et al. 2012; Siap et al. 2012; Zhu and Wang 2011). More recently, linear codes over the non-chain ring *Z*_4_ + *uZ*_4_, where *u*^2^ = 0, have been explored in Yildiz and Karadenniz (2014). The authors defined a linear Gray map from *Z*_4_ + *uZ*_4_ to *Z*_4_^2^ and a non-linear Gray map from *Z*_4_ + *uZ*_4_ to (*F*_2_ + *uF*_2_)^2^, and used them to successfully construct formally self-dual codes over *Z*_4_ and good non-linear codes over *F*_2_ + *uF*_2_. In Yildiz and Aydin (2014), the structure of cyclic codes over *Z*_4_ + *uZ*_4_ was determined and many new linear codes over *Z*_4_ were obtained from them. Motivated by the works in Yildiz and Aydin (2014) and Yildiz and Karadenniz (2014), we focus on constacyclic codes over *Z*_4_ + *uZ*_4_ and intend to construct good binary codes from such codes.

The ring *Z*_4_ + *uZ*_4_ is a finite commutative ring with characteristic 4, where *u*^2^ = 0. The purpose of this paper is to investigate a class of constacyclic codes over this ring, that is, (1 + *u*)-constacyclic codes over *Z*_4_ + *uZ*_4_. Constacyclic codes over finite commutative rings were first introduced by Wolfmann (1999), where it was proved that the binary image of a linear negacyclic code over *Z*_4_ is a binary cyclic code (not necessarily linear). In Kai et al. (2012), the authors introduced a composite Gray map from *F*_2_ + *uF*_2_ + *vF*_2_ + *uvF*_2_ to *F*_2_^4^ and proved that the image of a (1 + *u*)-constacyclic code of length n over *F*_2_ + *uF*_2_ + *vF*_2_ + *uvF*_2_ under the Gray map is a distance invariant binary quasi-cyclic code of index 2 and length 4*n*. It is known that the structure of *Z*_4_ + *uZ*_4_ is similar to that of *F*_2_ + *uF*_2_ + *vF*_2_ + *uvF*_2_. It is natural to ask if there exists a Gray map such that the Gray image of a linear code over *Z*_4_ + *uZ*_4_ has good structure. For this, we introduce a new Gray map from *Z*_4_ + *uZ*_4_ to *Z*_4_, and explore the images of (1 + *u*)-constacyclic codes over *Z*_4_ + *uZ*_4_ under this Gray map.

## (1 + *u*)-Constacyclic codes over *Z*_4_ + *uZ*_4_

Throughout this paper, let *R* denote the ring *Z*_4_ + *uZ*_4_ with *u*^2^ = 0. Any element in *R* can be written as *a* + *bu*, where *a*, *b* ∊ *Z*_4_. The element *a* + *bu* is a unit in *R* if and only if *a* is a unit in *Z*_4_. The ring *R* is a local Frobenius ring, but not a finite chain ring. It has a total of 7 ideals given by $I_{0} = \{ 0\} \subseteq I_{2u} = 2u(Z_{4} + uZ_{4} ) = \{ 0,2u\} \subseteq I_{u} ,I_{2} ,I_{2 + u} \subseteq I_{2,u} \subseteq I_{1} = Z_{4} + uZ_{4}$, where

$$ \begin{aligned} I_{u} & = u(Z_{4} + uZ_{4} ) = \{ 0,u,2u,3u\} , \\ I_{2} & = 2(Z_{4} + uZ_{4} ) = \{ 0,2,2u,2 + 2u\} , \\ I_{2 + u} & = (2 + u)(Z_{4} + uZ_{4} ) = \{ 0,2 + u,2u,2 + 3u\} , \\ I_{2,u} & = \{ 0,2,u,2u,3u,2 + u,2 + 2u,2 + 3u\} . \\ \end{aligned} $$

A code over *R* of length *n* is a nonempty subset of *R*_*n*_, and a code is linear over *R* of length *n* if it is an *R*-submodule of *R*_*n*_. For some fixed unit *λ* ∊ *R*, the *λ*-constacyclic shift *τ* on *R*_*n*_ is the shift *τ*(*c*_0_, *c*_1_, …, *c*_*n*−1_) = (*λc*_*n*−1_, *c*_0_, …, *c*_*n*−2_). A linear code C of length *n* over *R* is λ-constacyclic if the code is invariant under the λ-constacyclic shift *τ*. We identify the code-word *c* = (*c*_0_, *c*_1_, …, *c*_*n*−1_) with its polynomial representation *c*(*x*) = *c*_0_ + *c*_1_*x* + ··· + *c*_*n*−1_*x*^*n*−1^. Then *xc*(*x*) corresponds to a *λ*-constacyclic shift of *c*(*x*) in the ring *R*[*x*]/(*x*^*n*^ − *λ*). Thus, *λ*-constacyclic codes of length *n* over *R* can be identified as ideals in the ring *R*[*x*]/(*x*^*n*^ − *λ*). From the above discuss, we have the following result.

### **Proposition 1**

*A subset C of R*_*n*_*is a linear cyclic code of length n if and only if C is an ideal of*${{A_{n} = R[x]} \mathord{\left/ {\vphantom {{A_{n} = R[x]} {(x^{n} - 1)}}} \right. \kern-0pt} {(x^{n} - 1)}}$*. A subset C of R*_*n*_*is a linear*$(1 + u)$-*constacyclic code of length n over R if and only if C is an ideal of*${{B_{n} = R[x]} \mathord{\left/ {\vphantom {{B_{n} = R[x]} {(x^{n} - 1 - u)}}} \right. \kern-0pt} {(x^{n} - 1 - u)}}$.

Now, we determine a set of generators of (1 + *u*)-constacyclic codes for an arbitrary length over *R*. We begin by recalling a unique set of generators for cyclic codes over *Z*_4_.

### **Lemma 2**

[cf. Abualrub and Siap (2006), Theorem 6] *Let C be a cyclic code of length n over Z*_*4*_*. Then*

1. *If n is odd then*$C = \langle g(x),2a(x)\rangle = \langle g(x) + 2a(x)\rangle$*, where*$g(x),a(x)$*are binary polynomials with*$a(x)\left| {g(x)} \right.\left| {(x^{n} - 1)} \right.\bmod 2$.
2. *If n is even then*
  2.1 *If*$g(x) = a(x)$*, then*$C = \langle g(x) + 2p(x)\rangle$*, where*$g(x),p(x)$*are binary polynomials with*$g(x)\left| {(x^{n} - 1)} \right.\bmod 2$*, and*$g(x)\left| {p(x)\frac{{(x^{n} - 1)}}{g(x)}} \right.$,
  2.2 $C = \langle g(x) + 2p(x),2a(x)\rangle$*, where*$g(x),a(x)$*and*$p(x)$*are binary polynomials with*$a(x)\left| {g(x)} \right.\left| {(x^{n} - 1)} \right.\bmod 2$, $a(x)\left| {p(x)\frac{{(x^{n} - 1)}}{g(x)}} \right.$*and*$\deg g(x) > \deg a(x) > \deg p(x)$.

For a linear code *C* of length *n* over *R*, we can denote two linear codes of length *n* over *Z*_4_ as follows:

1. The torsion code *Tor*(*C*) = {*x* ∊ *Z*_4_^*n*^|*ux* ∊ *C*},
2. The residue code ${\text{Res}}(C) = \{ x \in Z_{4}^{n} \left| {\exists y \in Z_{4}^{n} :x + uy \in C} \right.\}$.

Consider the homomorphism $\varphi :R \to Z_{4}$ defined by $\varphi (a+ub)=a$. The map $\varphi$ extends naturally to a ring homomorphism $\varphi :R_{n} \to Z_{4} (n) = \frac{{Z_{4} [x]}}{{(x^{n} - 1)}}$ defined by

$$ \varphi (c_{0} + c_{1} x + \cdots ,c_{n - 1} x^{n - 1} ) = \varphi \left( {c_{0} } \right) + \varphi \left( {c_{1} } \right)x + \cdots + \varphi \left( {c_{n - 1} } \right)x^{n - 1} . $$

Acting $\varphi$ on *C* over *R*, we define a ring homomorphism

$$ \varphi :C \to {\text{Res}}(C),\varphi (a + ub) = a\quad {\text{where}}\;\,a,b \in Z_{4} . $$

We can easily obtain that $Ker \varphi$ ≅ *Tor*(*C*) and $\varphi$(*C*) = *Res*(*C*). By the first isomorphism theorem of finite groups, we have $\left| C \right| = \left| {Tor(C)} \right|\left| {{\text{Res}}(C)} \right|$. It is obvious that the image of *C* under the map $\varphi$ is a cyclic code of length *n* over *Z*_4_. Combining the above discussion with Lemma 2, we can obtain the set of generators for cyclic codes of length *n* over *R*.

### **Theorem 3**

*Let C be a*$(1 + u)$-*constacyclic code of length n over R*. *Then*

1. *If n is odd then*$C = \langle g_{1} (x) + 2a_{1} (x) + ub(x),u(g_{2} (x) + 2a_{2} (x))\rangle$*, where*$b(x)$*is a polynomial in*$Z_{4} [x]$*and*$g_{i} (x),a_{i} (x)$*are binary polynomials with*$a_{i} (x)\left| {g_{i} (x)} \right.\left| {(x^{n} } \right. - 1)\bmod 2\;for\;i = 1,2$.
2. *If n is even then*
  2.1 *If*$g_{i} (x) = a_{i} (x)$*, then*$C = \langle g_{1} (x) + 2p_{1} (x) + ud(x),u(g_{2} (x) + 2p_{2} (x))\rangle$*, where*$d(x)$*is a polynomial in*$Z_{4} [x]$, $g_{i} (x),p_{i} (x)$*are binary polynomials with*$g_{i} (x)\left| {(x^{n} } \right. - 1)\bmod 2\;$*and*$g_{i} (x)\left| {p_{i} (x)\frac{{(x^{n} - 1)}}{{g_{i} (x)}}} \right.,for\;i = 1,2$;
  2.2 $C = \langle g_{1} (x) + 2p_{1} (x) + ue_{1} (x),2a_{1} (x) + ue_{2} (x),ug_{2} (x) + 2up_{2} (x),2a_{2} (x)\rangle$*, where*$e_{i} (x)$*is a polynomial in*$Z_{4} [x]$*, and*$g_{i} (x),a_{i} (x),p_{i} (x)$*are binary polynomials with*$a_{i} (x)\left| {g_{i} (x)} \right.\left| {(x^{n} } \right. - 1)\bmod 2\;$, $a_{i} (x)\left| {p_{i} (x)\frac{{(x^{n} - 1)}}{{g_{i} (x)}}} \right.$*and*$\deg g_{i} (x) > \deg a_{i} (x) > \deg p_{i} (x)$,$for\;i = 1,2$.

### *Proof*

We only give the proof of the part (1), and the proof of the part (2) is similar.

Assume that *n* is odd. Let *C* be a (1 + *u*)-constacyclic code of length *n* over *R*. Then the image of *C* under the map $\varphi$ is Res(*C*), which is a cyclic code of length *n* over *Z*_4_. By Lemma 2, we have $\varphi$(*C*) = 〈*g*_1_(*x*) + 2*a*_1_(*x*)〉, where *g*_1_(*x*), *a*_1_(*x*) are binary polynomials with $a_{1} (x)\left| {g_{1} (x)} \right.\left| {(x^{n} } \right. - 1)\bmod 2\;$. Thus, there exists *b*(*x*) ∊ *Z*_4_[*x*] such that *g*_1_(*x*) + 2*a*_1_(*x*) + *ub*(*x*) ∊ *C*.

Furthermore, note that $Ker \varphi$ is a cyclic code of length *n* over *Z*_4_ + *uZ*_4_, so $Ker \varphi$ = *u* 〈*g*_2_(*x*) + 2*a*_2_(*x*)〉, where *g*_2_(*x*), *a*_2_(*x*) are binary polynomials with $a_{2} (x)\left| {g_{2} (x)} \right.\left| {(x^{n} } \right. - 1)\bmod 2\;$. Hence, 〈*g*_1_(*x*) + 2*a*_1_(*x*) + *ub*(*x*), *u*(*g*_2_(*x*) + 2*a*_2_(*x*))〉 ⊆ *C*.

On the other hand, for any *f*(*x*) = *f*_1_(*x*) + *uf*_2_(*x*) ∊ *C*, where *f*_*i*_(*x*) ∊ *Z*_4_[*x*], *for i* = 1, 2, it is obvious that *f*_1_(*x*) ∊ $\varphi$(*C*). Hence,

$$ \begin{aligned} f(x) & = f_{1} (x) + uf_{2} (x) \\ & = m(x)(g_{1} (x) + 2a_{1} (x)) + uf_{2} (x) \\ & = m(x)(g_{1} (x) + 2a_{1} (x) + ub(x)) + u(f_{2} (x) - m(x)b(x)) \\ \end{aligned} $$

Since *u*(*f*_2_(*x*) − *m*(*x*)*b*(*x*)) ∊ $Ker \varphi$, we have

$$ f(x) \in \langle g_{1} (x) + 2a_{1} (x) + ub(x),u\left( {g_{2} (x) + 2a_{2} (x)} \right)\rangle . $$

This shows that *C* ⊆ 〈*g*_1_(*x*) + 2*a*_1_(*x*) + *ub*(*x*), *u*(*g*_2_(*x*) + 2*a*_2_(*x*))〉.

Thus, *C* = 〈*g*_1_(*x*) + 2*a*_1_(*x*) + *ub*(*x*), *u*(*g*_2_(*x*) + 2*a*_2_(*x*))〉.□

## Gray images of (1 + *u*)-constacyclic codes over *R*

### A new Gray map

Recall that the Gray map $\phi _{1}$ from *Z*_4_ to *F*_2_^2^ is defined as $\phi _{1}$(*z*) = (*q*, *q* + *r*) where *z* = *r* + 2*q* with *r*, *q* ∊ *F*_2_. The map $\phi _{1}$ can be extended to *Z*_4_^*n*^ as follows:

$$ \begin{aligned} \phi_{1} & :Z_{4}^{n} \to F_{2}^{2n} \\ & \;\;(z_{0} ,z_{1} , \ldots ,z_{n - 1} ) \to (q_{0} ,q_{1} , \ldots ,q_{n - 1} ,q_{0} + r_{0} ,q_{1} + r_{1} , \ldots ,q_{n - 1} + r_{n - 1} ) \\ \end{aligned} $$

where *z*_*i*_ = *r*_*i*_ + 2*q*_*i*_ with *r*_*i*_, *q*_*i*_ ∊ *F*_2_*for* 0 ≤ *i* ≤ *n* − 1. It is known that $\phi _{1}$ is a distance-preserving map from *Z*_4_^*n*^ (Lee distance) to *F*_2_^2*n*^ (Hamming distance).

Now, we define a map $\phi _{2}$ from *R*^*n*^ to *Z*_4_^4*n*^. First note that each element *c* ∊ *R* can be expressed as *c* = *a* + *ub*, where *a*, *b* ∊ *Z*_4_. The map $\phi _{2}$ is defined as

$$ \phi_{2} (c) = \left( {b + 3a,b + 2a,b + a,b} \right). $$

Clearly, this map can be also extended to *R*^*n*^ as follows:

$$ \begin{aligned} \phi_{2} & :R^{n} \to Z_{4}^{4n} \\ & \;\;(c_{0} ,c_{1} , \ldots ,c_{n - 1} ) \to (b_{0} + 3a_{0} ,b_{1} + 3a_{1} , \ldots ,b_{n - 1} + 3a_{n - 1} ,b_{0} + 2a_{0} ,b_{1} \\ & \;\; + 2a_{1} , \ldots ,b_{n - 1} + 2a_{n - 1} ,b_{0} + a_{0} ,b_{1} + a_{1} , \ldots ,b_{n - 1} + a_{n - 1} ,b_{0} ,b_{1} , \ldots ,b_{n - 1} ) \\ \end{aligned} $$

where *c*_*i*_ = *a*_*i*_ + *ub*_*i*_ with *a*_*i*_, *b*_*i*_ ∊ *Z*_4_*for* 0 ≤ *i* ≤ *n* − 1.

It is well-known that the homogeneous weight has many applications for codes over finite rings and provides a good metric for the underlying ring in constructing superior codes. Next, we define a homogeneous weight on *R*. We first recall the definition of the homogeneous weight on a finite ring *K*.

#### **Definition 4**

*[cf. *Greferath and O’Sullivan (2004)*, Definition 1.1]* A real-valued function w on the finite ring K is called a (left) homogeneous weight if w(0) = 0 and the following is true:

1. For all x, y ∊ K, Kx = Ky implies that w(x) = w(y) holds.
2. There exists a real number γ such that ∑_y∊Kx_ w(y) = γ|Kx| for all $x \in K\backslash \{ 0\}$.

Right homogenous weight is defined accordingly. If a weight is both left homogenous and right homogeneous, we call it simply as a homogeneous weight.

For any element *c* = *a* + *ub* ∊ *R*, we assign the weight, denoted by *w*_hom_(*c*), as *w*_*L*_(*b* + 3*a*, *b* + 2*a*, *b* + *a*, *b*), i.e., *w*_hom_(*c*) = *w*_*L*_(*b* + 3*a*, *b* + 2*a*, *b* + *a*, *b*). By simple calculation, we can obtain the weight of any element *x* = *a* + *ub* ∊ *R* as follows:

$$ w_{\hom } (x) = \left\{ {\begin{array}{*{20}l} {0,} \hfill &\quad {x = 0} \hfill \\ {8,} \hfill &\quad {x = 2u} \hfill \\ {4,} \hfill &\quad {\text{otherwise} } \hfill \\ \end{array} } \right. $$

It is easy to verify that the weight defined above meets the conditions of Definition 4, hence it is actually a homogeneous weight on *R*. The homogeneous distance of a linear code over *R*, denoted by *d*_hom_(*C*), is defined as the minimum homogeneous weight of nonzero codewords of *C*. It can be checked that the map $\phi _{2}$ is a distance-preserving map from *R*^*n*^ (homogeneous distance) to *Z*_4_^4*n*^ (Lee distance). Using the maps $\phi _{1}$ and $\phi _{2}$, we can define a composite map $\phi :R \to F_{2}^{8} \;\text{as} \;\phi = \phi_{1} \phi_{2}$. Thus, we have obtained three distance-preserving maps as follows:

$$ \begin{aligned} \phi_{1} & :\left( {Z_{4}^{n} ,\,{\text{Lee}}\;{\text{distance}}} \right) \to \left( {F_{2}^{2n} ,\,{\text{Hamming}}\;{\text{distance}}} \right), \\ \phi_{2} & :\left( {R^{n} ,{\text{homogeneous}}\;{\text{distance}}} \right) \to \left( {Z_{4}^{4n} ,\;{\text{Lee}}\,{\text{distance}}} \right), \\ \phi & :\left( {R^{n} ,\,{\text{homogeneous}}\;{\text{distance}}} \right) \to \left( {F_{2}^{8n} ,\;{\text{Hamming}}\;{\text{distance}}} \right). \\ \end{aligned} $$

### Gray images of (1 + u)-constacyclic codes

#### **Lemma 5**

*Let *$\phi _{2}$*be defined as above. Let τ be the *$(1 + u)$-*constacyclic shift on R*^*n*^*and σ be the cyclic shift on Z*_*4*_^*4n*^*. Then*$\phi _{2} \tau  = \sigma \phi _{2}$.

#### *Proof*

Let *c* = (*c*_0_, *c*_1_, …, *c*_*n*−1_) ∊ *R*^*n*^. Let *c*_*i*_ = *a*_*i*_ + *ub*_*i*_ where *a*_*i*_, *b*_*i*_ ∊ *Z*_4_*for* 0 ≤ *i* ≤ *n* − 1. From definitions, we have

$$ \begin{aligned} \phi_{2} (c) & = ({\text{b}}_{0} + 3a_{0} ,{\text{b}}_{1} + 3a_{1} , \ldots ,{\text{b}}_{n - 1} + 3a_{n - 1} ,{\text{b}}_{0} + 2a_{0} ,{\text{b}}_{1} + 2a_{1} , \ldots , {\text{b}}_{n - 1}\\ & \quad  + 2a_{n - 1} ,{\text{b}}_{0} + a_{0} ,{\text{b}}_{1} + a_{1} , \ldots ,{\text{b}}_{n - 1} + a_{n - 1} ,{\text{b}}_{0} ,{\text{b}}_{1} , \ldots ,{\text{b}}_{n - 1} ) \\ \end{aligned} $$

Hence,

$$ \begin{aligned} \sigma \phi_{2} (c) & = ({\text{b}}_{n - 1} ,{\text{b}}_{0} + 3a_{0} ,{\text{b}}_{1} + 3a_{1} , \ldots ,{\text{b}}_{n - 1} + 3a_{n - 1} ,{\text{b}}_{0} + 2a_{0} ,{\text{b}}_{1} + 2a_{1} , \ldots , {\text{b}}_{n - 1}\\ & \quad  + 2a_{n - 1} ,{\text{b}}_{0} + a_{0} ,{\text{b}}_{1} + a_{1} , \ldots ,{\text{b}}_{n - 1} + a_{n - 1} ,{\text{b}}_{0} ,{\text{b}}_{1} , \ldots ,{\text{b}}_{n - 2} ) \\ \end{aligned} $$

On the other hand,

$$ \begin{aligned} \tau (c) & = ((1 + u)c_{n - 1} ,c_{0} ,c_{1} , \ldots ,c_{n - 2} ) \\ & = (a_{n - 1} + u(a_{n - 1} + b_{n - 1} ),a_{0} + ub_{0} , \ldots ,a_{n - 2} + ub_{n - 2} ) \\ \end{aligned} $$

Thus,

$$ \begin{aligned} \phi_{2} \tau (c) & = ({\text{b}}_{n - 1} ,{\text{b}}_{0} + 3a_{0} ,{\text{b}}_{1} + 3a_{1} , \ldots ,{\text{b}}_{n - 1} + 3a_{n - 1} ,{\text{b}}_{0} + 2a_{0} ,{\text{b}}_{1} + 2a_{1} , \ldots ,{\text{b}}_{n - 1} \\ & \quad + 2a_{n - 1} ,{\text{b}}_{0} + a_{0} ,{\text{b}}_{1} + a_{1} , \ldots ,{\text{b}}_{n - 1} + a_{n - 1} ,{\text{b}}_{0} ,{\text{b}}_{1} , \ldots ,{\text{b}}_{n - 2} ) \\ \end{aligned} $$

The result follows.□

#### **Theorem 6**

*A linear code C of length n over R is a*$(1 + u)$-*constacyclic code if and only if *$\phi _{2}$(*C*) *is a cyclic code of length 4n over Z*_*4*_.

#### *Proof*

If *C* is a (1 + *u*)-constacyclic code, then using Lemma 5 we have

$$ \sigma \left( {\phi_{2} (C)} \right) = \phi_{2} \left( {\tau (C)} \right) = \phi_{2} (C). $$

Hence, $\phi _{2}$(*C*) is a cyclic code of length 4*n* over *Z*_4_.

Conversely, if $\phi _{2}$(*C*) is a cyclic code of length 4*n* over *Z*_4_, then using Lemma 5 again we get $\phi _{2}$(*τ*(*C*)) = *σ*($\phi _{2}$(*C*)) = $\phi _{2}$(*C*).

Note that $\phi _{2}$ is injection, so *τ*(*C*) = *C*.

Thus, we immediately have the following result.□

#### **Corollary 7**

*The image of a*$(1 + u)$-*constacyclic code of length n over R under the map *$\phi _{2}$*is a distance invariant cyclic code of length 4n over Z*_*4*_.

Let *σ* be the cyclic shift. For any positive integer *s*, let *σ*_*s*_ be the quasi-shift given by

$$ \sigma_{s} (a^{(1)} \left| {a^{(2)} } \right.\left| { \cdots \left| {a^{(s)} } \right.} \right.) = (\sigma (a^{(1)} )\left| {\sigma (a^{(2)} )} \right.\left| { \cdots \left| {\sigma (a^{{({\text{s}})}} )} \right.} \right.) $$

where *a*^(1)^, *a*^(2)^, …, *a*^(*s*)^ ∊ *F*_2_^2*n*^ and “|”denotes the usual vector concatenation. A binary quasi-cyclic code *C* of index *s* and length 2*ns* is a subset of (*F*_2_^2*n*^)^*s*^ such that *σ*_*s*_(*C*) = *C*.

#### **Lemma 8**

*Let *$\phi$*be defined as above and let τ be the* $(1 + u)$-*constacyclic shift on R*^*n*^*. Then * $\phi \tau  = \sigma _{4} \phi$.

#### *Proof*

Let *r* = (*r*_0_, *r*_1_, …, *r*_*n*−1_) ∊ *R*^*n*^. Let *r*_*i*_ = *a*_*i*_ + 2*b*_*i*_ + *uc*_*i*_ + 2*ud*_*i*_ where *a*_*i*_, *b*_*i*_, *c*_*i*_, *d*_*i*_ ∊ *F*_2_*for* 0 ≤ *i* ≤ *n* − 1. Then we have

$$ \begin{aligned} \phi (r) & = (a_{0} + b_{0} + d_{0} , \ldots ,a_{n - 1} + b_{n - 1} + d_{n - 1} ,a_{0} + d_{0} , \ldots ,a_{n - 1} + d_{n - 1} ,b_{0} + d_{0} , \ldots ,b_{n - 1}  \\ & \quad + d_{n - 1} , d_{0} , \ldots ,d_{n - 1} ,b_{0} + c_{0} + d_{0} , \ldots ,b_{n - 1} + c_{n - 1} + d_{n - 1} ,a_{0} + c_{0} + d_{0} , \ldots ,a_{n - 1} + c_{n - 1}  \\ & \quad + d_{n - 1} , a_{0} + b_{0} + c_{0} + d_{0} , \ldots ,a_{n - 1} + b_{n - 1} + c_{n - 1} + d_{n - 1} ,c_{0} + d_{0} , \ldots ,c_{n - 1} + d_{n - 1} ) \\ \end{aligned} $$

and so

$$ \begin{aligned} \sigma_{4} \phi (r) & = (a_{n - 1} + d_{n - 1} ,a_{0} + b_{0} + d_{0} , \ldots ,a_{n - 1} + b_{n - 1} + d_{n - 1} ,a_{0} + d_{0} , \ldots ,a_{n - 2}  \\ & \quad + d_{n - 2} , d_{n - 1} ,b_{0} + d_{0} , \ldots ,b_{n - 1} + d_{n - 1} ,d_{0} , \ldots ,d_{n - 2} ,a_{n - 1} + c_{n - 1} + d_{n - 1} ,b_{0} + c_{0} \\ & \quad + d_{0} , \ldots ,b_{n - 1} + c_{n - 1} + d_{n - 1} ,a_{0} + c_{0} + d_{0} , \ldots ,a_{n - 2} + c_{n - 2} + d_{n - 2} ,c_{n - 1}  \\ & \quad + d_{n - 1} , a_{0} + b_{0} + c_{0} + d_{0} , \ldots ,a_{n - 1} + b_{n - 1} + c_{n - 1} + d_{n - 1} ,c_{0} + d_{0} , \ldots ,c_{n - 2} + d_{n - 2} ) \\ \end{aligned} $$

On the other hand,

$$ \begin{aligned} \tau (r) & = ((1 + u)r_{n - 1} ,r_{0} ,r_{1} , \ldots ,r_{n - 2} ) \\ & = ((a_{n - 1} + 2b_{n - 1} ) + u(a_{n - 1} + c_{n - 1} ) + 2u(b_{n - 1} + d_{n - 1} ),a_{0} \\ & \quad  + 2b_{0} + uc_{0} + 2ud_{0} , \ldots ,a_{n - 2} + 2b_{n - 2} + uc_{n - 2} + 2ud_{n - 2} ) \\ \end{aligned} $$

Hence,

$$ \begin{aligned} \phi \tau (r) & = (a_{n - 1} + d_{n - 1} ,a_{0} + b_{0} + d_{0} , \ldots ,a_{n - 1} + b_{n - 1} + d_{n - 1} ,a_{0} + d_{0} , \ldots ,a_{n - 2}  \\ & \quad + d_{n - 2} , d_{n - 1} ,b_{0} + d_{0} , \ldots ,b_{n - 1} + d_{n - 1} ,d_{0} , \ldots ,d_{n - 2} ,a_{n - 1} + c_{n - 1} + d_{n - 1} ,b_{0} + c_{0} \\ & \quad + d_{0} , \ldots ,b_{n - 1} + c_{n - 1} + d_{n - 1} ,a_{0} + c_{0} + d_{0} , \ldots ,a_{n - 2} + c_{n - 2} + d_{n - 2} ,c_{n - 1} \\ & \quad + d_{n - 1} ,a_{0} + b_{0} + c_{0} + d_{0} , \ldots ,a_{n - 1} + b_{n - 1} + c_{n - 1} + d_{n - 1} ,c_{0} + d_{0} , \ldots ,c_{n - 2} + d_{n - 2} ) \\ \end{aligned} $$

This completes the proof.□

#### **Theorem 9**

*A linear code C of length n over R is a* $(1 + u)$-*constacyclic code if and only if *$\phi$(*C*) *is a binary quasi-cyclic code of index 4 and length 8n.*

#### *Proof*

If *C* is (1 + *u*)-constacyclic, then using Lemma 8 we have

$$ \sigma_{4} \left( {\phi (C)} \right) = \phi \left( {\tau (C)} \right) = \phi (C). $$

Hence, $\phi$(*C*) is a binary quasi-cyclic code of index 4 and length 8*n*. Conversely, if $\phi$(*C*) is a binary quasi-cyclic code of index 4 and length 8*n*, then using Lemma 8 again we get $\phi$(*τ*(*C*)) = *σ*_4_($\phi$(*C*)) = $\phi$(*C*).

Also, $\phi$ is injection, hence *τ*(*C*) = *C*.□

From Theorem 9 and the definition of the map $\phi$, we immediately have the following result.

#### **Corollary 10**

*The image of a* (1 + *u*)-*constacyclic code of length n over R under the map *$\phi$*is a distance invariant binary quasi-cyclic code of index 4 and length 8n.*

Now, we can construct some binary codes with good parameters based on the new Gray map.

#### *Example 11*

Consider (1 + *u*)-constacyclic codes over *Z*_4_ + *uZ*_4_ of length 3. In *F*_2_[*x*], *x*^3^ − 1=(*x* − 1)(*x*^2^ + *x* + 1).

1. In Theorem 3, we take *g*_1_(*x*) = *x*−1, *a*_1_(*x*) = 1, *b*(*x*) = 3*x*, and *g*_2_(*x*) = *a*_2_(*x*) = *x*^3^ − 1. Then, we obtain the (1 + *u*)-constacyclic code *C*_1_ over *R* of length 3 with generator polynomial (1 + *u*)*x* + 1. That is 〈(1 − *u*)*x* + 1〉 = 〈*x* + (1 + *u*)〉, It is easy to see that both Res(*C*_1_) and *Tor*(*C*_1_) have size 16. Moreover, *d*_hom_(*C*_1_) = 8. By Corollary 7, $\phi$_2_(*C*_1_) is a *Z*_4_-linear code of length 12 with size 256 and Lee distance 8. By Theorem 9, $\phi$(*C*_1_) is a binary quasi-cyclic code of index 4 and length 24. We find that $\phi$(*C*_1_) is a non-linear binary code with parameters (24, 256, 8). The code $\phi$(*C*_1_) attains the performance of the best-known binary linear code with the same parameters based on Grassl’s codetables (Grassl 2007).
2. In Theorem 3, $g_{1} (x) = x^{3} + 1,a_{1} (x) = 1,b(x) = 3,g_{2} (x) = x + 1,\text{and} \;a_{2} (x) = x + 1$. Then, we obtain the code *C*_2_ = 〈3*u*(*x* + 1)〉 = 〈*u*(*x* + 1)〉. Obviously, Res(*C*_2_) = {0} and *Tor*(*C*_2_) has size 4. By Corollary 7, $\phi$(*C*_2_) is a Z_4_-linear code of length 12 with size 4 and Lee distance 16. By Theorem 9, $\phi$(*C*_2_) is a binary quasi-cyclic code of index 4 and length 24. The code $\phi$(*C*_2_) is a linear binary code with parameters [24, 2, 16], which is optimal based on Grassl’s codetables (Grassl 2007).

## Conclusion

We study the structure of (1 + *u*)-constacyclic codes over *Z*_4_ + *uZ*_4_ of an arbitrary length, and obtain the examples of good binary codes from them. Our results show that a (1 + *u*)-constacyclic code of length n over *Z*_4_ + *uZ*_4_ under certain map is equivalent to a cyclic code of length 4*n* over *Z*_4_. Furthermore, we discuss the relation between (1 + *u*)-constacyclic codes of length *n* over *Z*_4_ + *uZ*_4_ and their binary Gray images. It would be interesting to study other constacyclic codes over *Z*_4_ + *uZ*_4_ and use them to construct more good codes over *Z*_4_ or *F*_2_.
